# International university students’ perspectives on mental health and help-seeking behaviours in China: An exploratory qualitative study

**DOI:** 10.1371/journal.pmen.0000559

**Published:** 2026-04-06

**Authors:** Magnus Michael Sichalwe, Minjie Chu, Grace Tavengana, Manas Ranjan Behara, Abdul Basit

**Affiliations:** 1 Division of Health, Nutrition and Social Welfare Services, Butiama District Council, Mara, Tanzania; 2 Department of Epidemiology, School of Public Health, Nantong University, Jiangsu, China; 3 School of Public Health, Kalinga Institute of Industrial Technology, Deemed to be a University, Bhubaneswar, India; 4 Department of Nursing and Rehabilitation Medicine, Nantong University, Jiangsu, China; National University of Singapore, SINGAPORE

## Abstract

Global mental health concerns are increasing, with international students particularly vulnerable due to cultural adjustment, academic stress, and social isolation. Despite growing awareness, stigma and limited knowledge continue to hinder help-seeking. This study explored international university students’ perceptions of mental health and help-seeking behaviours at Nantong University, China. This qualitative study employed purposive sampling to recruit 20 international students from Nantong University. Data were collected between 14^th^ January and 4^th^ February 2025 through in-depth, semi-structured interviews, which were audio-recorded and transcribed verbatim. Thematic analysis was used to identify key patterns in participants’ mental health perceptions and help-seeking experiences. Ethical protocols were strictly followed, including informed consent and confidentiality safeguards. Following thematic analysis, five main themes and 16 subthemes emerged, covering mental health literacy, challenges faced, coping strategies, barriers to seeking professional help, and recommendations for improving support. Key findings revealed diverse understandings of mental health, significant stressors like isolation and academic pressure, reliance on peer support, and barriers such as stigma, privacy concerns, and lack of awareness. Recommendations included ensuring confidentiality, enhancing cultural competence, and increasing accessibility to mental health services. International university students face mental health challenges and need accessible, confidential, and culturally sensitive support to reduce stigma and encourage help-seeking.

## Introduction

In 2023, approximately 4.5 million tertiary-level students studied abroad, with the majority originating from Asian countries, followed by African nations, forming the largest groups enrolled in higher education programmes globally [1]. Reflecting this trend, China hosted 592,185 international students in 2022, as the Chinese Ministry of Education reported. Most of these students came from Asia (295,043), followed by Africa (81,562), Europe (73,618), the Americas (35,733), and Oceania (6,229) [2]. Psychological distress and mental health disorders are growing concerns among university students, with challenges often more pronounced in international students [3]. Studies in China have found that 59.7% [4] and 56.8% [5] of international university students experience depression.

Studies show that both international and local tertiary students often avoid seeking treatment for mental health issues, resulting in significant impairment [5,6]. However, this reluctance is particularly pronounced among international students, who frequently hesitate to seek formal help for their mental health problems [7]. A recent Victorian Coronial investigation into the suicide of a Chinese student in Australia highlighted that failure to seek help for worsening mental health was a common factor in several international student suicides [8]. This is evidence that international students often delay seeking assistance until their mental health issues have reached a crisis point [9].

Although the Chinese government has prioritised mental health education and established university counselling services, many students in need of support refrain from using on-campus resources [10]. In 2018, the Ministry of Education of the People’s Republic of China (MOE) introduced new guidelines to promote mental health in universities, setting a counsellor-to-student ratio of no less than 1:4000 and requiring at least two on campus counsellors per institution [11]. Despite universities hiring part time counsellors to meet these standards, the availability of support still falls short of the students’ needs [12].

International students in China face language barriers and limited social connections, increasing their risk of anxiety and depression [13]. Early detection is crucial, as delayed treatment can worsen outcomes [14]. With most research focusing on domestic students, this qualitative study at Nantong University (NTU) explored international university students’ views on professional support, mental health literacy, and help-seeking behaviours to identify challenges and opportunities for improvement.

## Methodology

### Ethics statement

Ethical approval for this study was granted by the NTU Institutional Review Board (NTU/IRB/PHPM/2025/011). Written informed consent was obtained from all participants. Confidentiality was ensured through anonymised transcripts and secure data storage. Participation was voluntary, and individuals could withdraw at any time without penalty. Interviews were held at times convenient for participants to support open discussion. Ethical standards and methodological rigour were maintained throughout the study.

### Study design and setting

An exploratory qualitative design was used to examine international university students’ perspectives on mental health and help-seeking at NTU, China, offering in-depth insights into their challenges, barriers, and suggestions. Founded in 1912 in Nantong, Jiangsu Province, NTU is a comprehensive institution offering undergraduate to doctoral programmes. With students from over 50 countries, it provides language training, cultural integration support, and modern facilities, including research labs and libraries.

### Study population

The study included NTU international university students aged 18 and above, representing diverse backgrounds, to provide insights into mental health and help-seeking behaviours.

### Sample size determination and sampling technique

Sample size was determined by the concept of information power rather than by the pursuit of saturation. In line with Braun and Clarke’s [15] critique of saturation as a mechanical endpoint in qualitative research, data collection was guided by ongoing analytic judgement about the adequacy, richness, and relevance of the data in relation to the study aims. Drawing on Malterud et al [16], information power was considered in terms of the clarity of the research aim, the specificity of the sample, the quality of the interviews, and the analytic approach. On this basis, 20 interviews were judged to provide sufficient depth and breadth to support a nuanced understanding of international students’ perceptions of mental health and help-seeking behaviours.

Purposive sampling was used to recruit 20 international university students enrolled at NTU. Participants were international students aged 18 years or older, able to communicate in English, and willing to take part in a single in-person interview lasting approximately 25–30 minutes. The inclusion of students from diverse academic backgrounds and cultural contexts, alongside varied experiences of mental health, strengthened the information power of the dataset and ensured that the data were adequate to capture meaningful patterns and support the study’s conclusions.

### Theoretical foundation

This study is informed by Stress and Coping Theory (Lazarus and Folkman) [17] and Mental Health Literacy Theory [18]. Stress and Coping Theory explains how international students appraise academic, social, and financial stressors and how these appraisals shape coping responses, including self-reliance, peer support, and help-seeking behaviours. Mental Health Literacy Theory focuses on knowledge and beliefs that support recognition and management of mental health problems, providing a lens to examine students’ understanding of mental health, symptom interpretation, and engagement with professional services. These frameworks guided the study’s conceptual framework, data collection, and analysis, enabling examination of mental health knowledge, coping strategies, and barriers to care within the study context while allowing cautious consideration of transferable mechanisms relevant to similar international student populations.

### Data collection methods

Data were collected through in-depth, semi-structured interviews to explore international university students’ perceptions of mental health and help-seeking behaviours. All interviews were conducted in English, as this is the primary medium of instruction for the participants. Interviews were audio-recorded and transcribed verbatim. Field notes were used to capture non-verbal cues and contextual details. A trained research assistant with mental health expertise supported data collection following a three-day training programme that covered qualitative interviewing techniques, ethical considerations, confidentiality, probing strategies, and accurate note-taking in a cross-cultural context. A pretest with two students was conducted to refine the interview guide, with pretest data excluded from the main analysis. Data collection took place between 14th January and 4th February 2025.

### Data analysis

Interviews were audio-recorded, transcribed verbatim, and translated into English where necessary. Data were analysed using Braun and Clarke’s reflexive thematic analysis [15], which was selected for its suitability in exploring and interpreting shared patterns of meaning across participants’ narratives in relation to perceptions of mental health and help-seeking behaviours. This analytic approach aligned with the study’s qualitative design and exploratory aim by allowing flexibility while maintaining analytic rigour. The analysis followed the six phases of thematic analysis: familiarisation with the data, initial coding, theme development, theme review, theme definition, and reporting. During coding, data segments were labelled descriptively, then compared and grouped based on conceptual similarity. Overlapping or closely related codes were discussed and refined through iterative team discussions to ensure that themes captured distinct, meaningful patterns while acknowledging conceptual relationships. Interpretive decisions, including merging or differentiating themes, were documented transparently, with reflexivity maintained throughout to minimise researcher bias. Final themes were clearly defined and illustrated with participant quotes, ensuring both analytic depth and transparency in representing international students’ experiences.

#### Reflexivity and researcher positioning.

Reflexivity was actively maintained throughout the research process to enhance analytic transparency and interpretive rigor. The lead researcher’s positionality as an international student familiar with cross-cultural academic environments was acknowledged as potentially shaping participant interactions, data interpretation, and theme development [19]. To critically engage with this influence, a reflexive journal was maintained to document assumptions, evolving interpretations, and key analytic decisions during data collection and analysis [20]. Interviews were conducted with openness and neutrality to minimise the imposition of prior experiences on participants’ accounts. Analytic decisions, including the refinement and development of codes and themes, were discussed within the research team and systematically documented, ensuring that researcher influence was acknowledged, examined, and managed throughout the study [15].

#### Trustworthiness of the study.

Trustworthiness was ensured through strategies addressing credibility, dependability, confirmability, and transferability. Credibility was supported through in-depth, face-to-face interviews with international students at NTU, the use of a semi-structured interview guide, and careful transcription and verification of data [21]. Cross-case comparison across participants from diverse academic and cultural backgrounds further strengthened the credibility of the findings. Dependability was enhanced by maintaining a transparent audit trail documenting data collection procedures and analytic decisions [22]. Confirmability was supported through systematic documentation of coding and theme development and the inclusion of verbatim quotations to ground interpretations in participants’ accounts. Transferability was facilitated by providing rich contextual descriptions of the study setting and participants, enabling readers to assess the applicability of the findings to similar international student populations in higher education contexts [23].

## Results

### Demographic information

The study included 20 international students (12 males, 8 females) aged 20–30 from 18 countries. Most were Master’s students (13) in fields like Engineering, Business, and Public Administration, while 7 were Bachelor’s students in Pharmacy, Computer Science, and related fields. Participants had been in China for 1–5 years, with most in their early study years, offering diverse perspectives (Table 1).

**Table 1:** Demographic information of the study participants.

ID	Age	Gender	Country	Education level	Professional Major	Year of Study	Number of years in China
P1	22	F	Cameroon	Master	Transportation engineering	1	1
P2	23	M	Zimbabwe	Master	Engineering	3	4
P3	24	F	Türkiye	Bachelor	MBBS	1	2
P4	20	F	Namibia	Bachelor	Pharmacy	3	3
P5	26	M	Pakistani	Master	Rehabilitation Medicine	1	1
P6	28	F	Zambia	Master	International Business	2	2
P7	24	M	Afghanistan	Masters	Civil engineering	2	2
P8	22	M	Thailand	Bachelor	Mechanical engineering	2	2
P9	26	F	Timor-Ceste	Masters	International Business	1	2
P10	25	M	Botswana	Masters	International Business	1	3
P11	26	M	Zambia	Masters	Public administration	2	5
P12	22	M	Indonesia	Bachelor	Business administration	2	3
P13	30	M	Liberia	Master	Civil engineering	1	5
P14	28	M	Bangladesh	Master	Textile engineering	3	4
P15	27	M	Algeria	Master	Public administration	1	1
P16	24	F	Morocco	Bachelor	Pharmacy	2	2
P17	26	F	Nigeria	Bachelor	Computer science	3	3
P18	25	F	Bangladesh	Master	International Business	2	3
P19	22	F	Uganda	Bachelor	Business administration	1	1
P20	24	M	India	Master	Rehabilitation Medicine	2	2

#### Overview of themes and subthemes.

Four main themes and associated subthemes were identified from the interviews (Table 2). Theme 1, Mental health literacy, reflects students’ understanding of mental health, common disorders, contributing factors, signs, and impacts. Theme 2, Mental health-related challenges, highlights social isolation, substance use, and academic and financial stress. Theme 3, Approaches to managing challenges, captures reliance on peers, family, and limited professional support. Theme 4, Barriers to professional help, includes confidentiality concerns, stigma, limited service access, self-reliance, misrecognition of issues, and communication difficulties.

**Table 2:** Themes and Subthemes.

Theme	Subtheme	Description (Short Summary)
**Theme 1: Mental health literacy**	**Knowledge of the meaning of mental health**	Students described mental health as emotional regulation, daily functioning, wellbeing, or linked it to mental illness; some showed limited understanding.
	**Knowledge of common mental health problems**	Students mentioned disorders such as anxiety, OCD, PTSD, depression, stress, and schizophrenia, but often confused symptoms with conditions.
	**Knowledge of contributors to mental health problems**	Cited contributors included trauma, academic pressure, financial stress, isolation, environmental stressors, and separation from family.
	**Perceived signs and symptoms of mental health problems**	Reported signs included irritability, social withdrawal, reduced communication, overeating, substance abuse, and low self-confidence.
	**Knowledge of the impact of mental health on well-being**	Students recognised effects on academics, physical health, relationships, and risk of self-harm; some believed issues were manageable with support.
**Theme 2: Mental health-related challenges faced by international students**	**Social isolation and substance abuse**	Students experienced loneliness, difficulty connecting socially, and some turned to excessive drinking as a coping mechanism.
	**Stress and anxiety due to academic and financial pressures**	Academic workload, unfamiliar systems, scholarship pressure, and financial instability contributed to stress and anxiety.
**Theme 3: Approaches to managing mental health challenges**	**Reliance on peers, family, and social support**	Students primarily sought emotional support from friends and relied on family communication for stability.
	**Limited reliance on professional help**	Few students considered counselling or professional support; they recognised its value but rarely accessed it.
**Theme 4: Barriers to seeking professional mental health support**	**Confidentiality and privacy concerns**	Fear of breached confidentiality on campus discouraged students from seeking professional help.
	**Limited access to services and lack of awareness**	Students reported not knowing where services were located or that they existed; lack of seminars worsened awareness.
	**Stigma and cultural perceptions**	Cultural norms discouraged counselling, reinforcing self-reliance and avoidance of mental health services.
	**Proactive and self-reliant coping strategies**	Students preferred resolving issues independently or through peers rather than using formal services.
	**Misunderstandings or lack of recognition of mental health issues**	Mental health issues were often normalised or not recognised due to being invisible or viewed as “typical student stress.”
	**Communication challenges**	Language differences, tone misunderstandings, and cultural mismatch with counsellors hindered help-seeking.

OCD: Obsessive Compulsive Disorders. PTSD: Post Traumatic Stress Disorders.

### Mental health literacy

#### Knowledge of the meaning of mental health.

International students demonstrated varied understandings of mental health, reflecting both personal interpretations and gaps in knowledge. Some participants conceptualised mental health broadly, linking it to overall wellbeing, the mind-body connection, emotional regulation, and coping with daily pressures.

***“****Okay, for me, mental health is the overall well-being of human beings. It's the way of thinking that deals with the coping of people's behaviours, their thinking, their emotions, and, yeah, their overall well-being. It's like the well-being of people's emotions, thoughts, etc., in the whole package. Yeah, it's mental health”. (P*#*11, 26 years old, Male, Master's Student)*

Conversely, other participants associated mental health primarily with psychological problems, emotional instability, or mental illness, suggesting a more limited or problem-focused perspective. Some expressed difficulty articulating a definition, highlighting the concept’s complexity and possible gaps in awareness.

*“I’m not familiar with this topic, so I think it’s best to skip this question and move on to the next one”. (P*#*15, 27 years old, Male, Master’s Student)*

These patterns indicate that participants’ understanding of mental health ranges from holistic wellbeing to mental disorder, with some expressing uncertainty or limited knowledge, reflecting differences in prior exposure and conceptual framing.

#### Knowledge of common mental health problems.

Participants identified a variety of mental health problems, ranging from well-known psychiatric disorders to everyday psychological challenges. Some named specific conditions such as obsessive compulsive disorder (OCD), post traumatic stress disorder (PTSD), depression, and schizophrenia:

*"There are some anxiety disorders, like... what’s that one? There’s... is it OCD? Yes, OCD, something to do with people who like clean spaces. We call it OCD, Obsessive Compulsive Disorder. Yes, OCD, and PTSD. They’re also mood disorders, like depression, stress, and all. There’s this other condition... is it schizophrenia? Yeah! Schizophrenia.”* *(P#12, 22 years old, Male, Bachelor Student)*

Other participants conflated symptoms with disorders, describing experiences such as stress, overthinking, or emotional strain as standalone mental health problems rather than manifestations of underlying conditions.

*“Mmh.. there is the heart because I think when you stick too much information in your heart or you keep it for yourself you can have mental health issues, there is the brain, I think overthinking too is a form of mental health disturbance.” (P#1, 22 years old, Female, Master’s Student)*

These responses reveal two patterns: first, some participants have partial knowledge of clinical mental health conditions; second, there is a tendency to interpret symptoms as discrete problems. This highlights variability in understanding and the influence of personal experience or informal knowledge on how mental health is conceptualised.

#### Knowledge of contributors to mental health problems.

Participants identified a range of factors they perceive as contributing to mental health problems, including social and family environments, economic challenges, separation from loved ones, and broader societal stressors such as epidemics. Generally, participants perceive mental health problems as arising from a combination of personal, social, and environmental factors, highlighting the multifaceted nature of contributors and the interplay between individual experiences and contextual stressors.

*“As I told you before,... Like, the main impact is your workplace or the environment that is around you. Like, if you are a student, it will be your university. If you are doing a job somewhere, it will be your office or wherever you are. If you are in the home, then it will be affected by the home as well. Moreover, it is also connected to the financial.”* (P#7, 24 years old, Male, Master’s Student)

Other participants emphasised trauma, isolation, environmental stressors, and feelings of being misunderstood or having low self-confidence as influential:

*“Mainly, I would say trauma. Be it family trauma, be it tragic trauma like accidents, be it multiple sorts of trauma, that's one. So the trauma, environment, isolation, being misunderstood.”* (P#16, 24 years old, Female, Bachelor Student)

#### Perceived signs and symptoms of mental health problems.

Participants identified a range of behavioural and emotional signs they associate with mental health issues, reflecting both internal struggles and outward coping behaviours. Social withdrawal, reduced communication, and unusual quietness were frequently mentioned, suggesting that students recognise observable changes in engagement as potential indicators of distress:

*"I think self isolation is the biggest sign. I don't think I know any other ones. Apart from that bigger one, other small ones. People are talking less. Talking less. Talking less, going out less. All those things that eventually lead to self-isolation, I guess.”* *(P#4, 24 years old, Female, Master's Student)*

Unhealthy coping strategies were identified as key indicators of mental health challenges, with participants describing behaviours such as overeating and excessive alcohol or drug use as ways of managing emotional distress. Low self-confidence and negative behaviours were also highlighted, reflecting the belief that internal psychological struggles manifest through reduced self-worth and unhealthy actions:

“Some people just eat to try and cope, and also excessive substance use, whether alcohol or drugs… less confidence can also be a symptom, and unhealthy behaviour or bad actions too, these are signs, in my mind.” *(P#19, 22 years old, Female, Bachelor Student)*

Overall, participants’ accounts illustrate that perceived signs of mental health problems span behavioural, emotional, and social domains, reflecting an implicit understanding that internal distress often manifests externally, even if the conceptual link between symptoms and clinical conditions is not always precise.

#### Knowledge of the impact of mental health on well-being.

Participants generally recognised that mental health significantly influences overall well-being, affecting academic performance, relationships, work, and daily functioning. Some emphasized severe consequences, including physical deterioration and suicidal behaviour, reflecting an awareness of the potentially profound effects of untreated mental health problems:

*"Of course, if someone’s mental health is not okay, they might stop talking to others, skip meals, and lose their physical and mental stability. In such cases, what can they do in their life? They might even take their own life. They can commit suicide. As a human, if someone is not okay, they might end their life. This is the reality.”* *(P#8, 22 years old, Male, Bachelor Student)*

Conversely, other participants framed mental health challenges as manageable, particularly when strong social support is available. This perspective suggests that students perceive the impact of mental health as variable and context dependent:

*“Don't think so because it's not a big deal to overcome, so people can easily overcome their mental health sometimes, because as I mentioned before, if I get homesickness, I just call my family members and talk with them. So I think it's easier to overcome this mental health, especially for me. It's my opinion.”* (P#14, 28 years old, Male, Master's Student)

While some participants perceive mental health problems as having severe, life-altering consequences, others view their impact as mitigated by coping strategies and social support. This variation highlights differences in personal experiences, resilience, and access to resources in shaping perceptions of mental health outcomes.

### Mental health-related challenges faced by international students

#### Social isolation and substance abuse.

Participants identified social isolation as a major mental health challenge for international students, often leading to emotional distress and, in some cases, substance abuse such as excessive drinking. They described feelings of loneliness, difficulty forming connections, and a lack of support as key contributors.

*“Judging from my experiences with going outside, I would say, especially the substance abuse, like excessive drinking. Also, for females, isolation is too much. You know, sometimes you just go outside and see a new face that you've never seen before, but you're told this person has been here for like two years or more, and you wonder why… yeah, the isolation, the substance abuse. And also, I think some financial issues. Yeah”. (P*#*6, 28 years old, Female, Master’s Student)*

Participants’ accounts suggest that social isolation is not only an emotional burden but can also trigger maladaptive coping strategies. The responses indicate that the mental health impact of isolation is compounded by additional stressors, such as financial pressures and challenges in integrating into new social environments, underscoring the multidimensional nature of isolation-related mental health risks.

#### Stress and anxiety due to academic and financial pressures.

Participants identified stress and anxiety as major challenges, with the unfamiliar environment, academic demands, and fear of losing scholarships creating persistent pressure that hinders adaptation and affects well-being and academic performance.

*“I think anxiety… Many of us didn’t study in China before, so we’re not used to the environment. Even if you haven’t done anything wrong, constant notices about scholarship cancellations feel like daily threats. When I first arrived, I was extremely anxious, especially about opening WeChat, which was always full of negative news.” (P#5, 25 years old, Female, Master's Student)*

These accounts indicate that stress and anxiety among international students are closely linked to environmental and institutional pressures, suggesting that adaptation difficulties and uncertainty about academic standing intensify psychological strain.

### Approaches to managing mental health challenges

#### Reliance on peers, family, and social support.

International students shared various approaches to managing mental health struggles, emphasizing self-reliance, peer support, and external connections. Students said they relied on friends or fellow students who could relate to their experiences, finding comfort in shared understanding and mutual support as their primary coping strategies.

*“I will try to help by talking to her and helping her feel understood. Keeping things to ourselves can affect us a lot. If we create an opportunity to share, she might feel relief and less alone.” (P#1, 23 years old, Male, Master’s Student)*

Additionally, participants emphasized the importance of family support and maintaining connections with loved ones back home, which provided emotional stability and reassurance during difficult times.

*“I talk to people from home. Yeah, I feel they're going to understand me better than I'll be understood here. Even in the matter of an emergency, I will still talk with people back home” (P*#*18, 25 years old, Male, Master's Student)*

These findings suggest that international students’ coping strategies are socially oriented, relying on relational networks to buffer stress and foster a sense of belonging. Peer and family support appear to serve complementary roles; peers provide immediate shared experiences, while family offers emotional continuity and reassurance.

#### Limited reliance on professional help.

Few international students recognised the value of seeking professional mental health support, with most relying on friends, family, or self-coping strategies. Even when acknowledged as helpful, professional help was often seen as a last resort after informal coping mechanisms failed. While some students recognise professional support as beneficial, it appears underutilised, indicating limited awareness or reliance on formal mental health services alongside peer and family support.

*“I'm not a doctor to answer this question. But for a human, I have to... If it happens to me, I will go to the doctor and tell him the story. Maybe she or he can help me. And for my friend also, if I see this situation, I will invite him or I will take him to the doctor”. (P*#*6, 28 years old, Female, Master’s Student)*

### Barriers to seeking professional mental health

#### Confidentiality and privacy concerns.

International students identified confidentiality and privacy issues as key barriers to accessing campus mental health services. Participants expressed distrust of university authorities, fearing that personal information would not be adequately protected.

*“We feel our privacy is always breached here… How do we know certain people have been called for therapy? It's already out there.” (P#6, 28 years old, Female, Master’s Student)*

Others reported that concerns about confidentiality discouraged them from using available services, as they feared personal information might be shared without consent. This mistrust led some students to avoid seeking professional help altogether, even when they recognised a need for support, highlighting how privacy concerns can act as a significant barrier to accessing mental health resources:

*“Our privacy is not protected that much. One way or the other, someone will know what you're going through.” (P#18, 25 years old, Male, Master's Student)*

#### Limited access to services and lack of awareness.

International students identified limited access to and awareness of mental health services as major challenges. Many participants reported difficulties in finding or using on campus counselling services, often citing a lack of clear information and outreach. Others stated they were unaware that such services were available at all.

*“I’m not sure I can pinpoint where, but I think it’s somewhere on campus here. Yeah, one of these offices, but I don’t know the exact location.” (P*#*13, 30 years old, Male, Master's Student)*

Participants noted they were often unaware of available support until informed through informal channels and felt unprepared to face challenges, including mental health issues. They said the lack of seminars or guidance on coping strategies left them feeling unsupported as they adjusted to their new environment.

*“Firstly we've been here for some months now and we've never even had any seminar on how to deal with these issues, where even for the mental, even not just the mental health”. (P*#*10, 25 years old, Male, Master's Student)*

Both limited access to services and lack of information hinder international students’ use of mental health resources, while insufficient outreach and guidance contribute to isolation and greater vulnerability to stress and anxiety.

#### Stigma and cultural perceptions.

Participants reported that cultural stigma discouraged them from seeking professional mental health support. Counselling was often viewed negatively in their home communities, and when combined with privacy concerns and limited awareness, this perception created significant barriers to accessing help.

*“We need counselling because we’re here alone… But when we reach out, we’re told to ‘be careful’ and that they’re ‘not responsible.’” (P#8, 22 years old, Male, Bachelor Student)*

Cultural attitudes shape help-seeking behaviour, with stigma acting as a powerful deterrent alongside practical barriers, reinforcing reliance on informal coping strategies rather than professional support.

#### Proactive and self-reliant coping strategies.

Participants demonstrated a proactive and self-reliant approach to managing mental health, focusing on identifying the root causes of their issues and seeking solutions through social activities and peer support. International students revealed a tendency to rely on informal support systems, such as friends and personal coping strategies, rather than seeking professional help.

*“If it is depression, firstly, I will try to find the reason for being depressed, you know, understand what’s causing it. For mental health issues like depression, I can invite my friends to more social activities and try to focus on positive aspects of life, like exploring new things.” (P*#*3, 24 years old, Female, Bachelor Student)*

#### Misunderstandings or lack of recognition of mental health issues.

Participants said a major barrier to seeking professional help was a misunderstanding or a lack of awareness of mental health issues. They pointed out that stress, anxiety, and depression were often viewed as temporary or typical aspects of student life, which led to these problems being overlooked and left unaddressed.

*“I think in social life, if students are stressed and they have depression, people see it, like, as a normal thing. They are thinking if you are a student, you have a hard life because you are struggling with maybe studies or something. But I think it's completely wrong”. (P*#*3, 24 years old, Female, Bachelor Student)*

International students said mental health issues are often underestimated because they are not visibly apparent, leading others to dismiss or misunderstand their struggles. They noted that this lack of recognition or misclassification delays seeking professional help.

*“Because when you're sick on the outside, when you're physically sick, you can see it. Someone can tell you your leg is broken. But when you're feeling heartache, no one can see that. It's just you. So they're not going to take things they can't see seriously”. (P*#*5, 25 years old, Male, Master’s Student)*

Both cultural assumptions about student life and the invisible nature of mental health symptoms contribute to delays in help-seeking, reinforcing reliance on informal coping mechanisms rather than professional support.

#### Communication challenges.

Participants reported that language barriers, cultural differences, and difficulty expressing emotions in a non-native language hindered access to mental health services. Limited cultural understanding by counsellors, poor translations, and unclear communication created disconnects, increased stress, and discouraged help-seeking:

*“It’s confusing because they speak English, but it’s hard to understand their tone and meaning. The way they speak can sound harsh, even if it’s normal for them. Notices often seem translated from Chinese, leading to misunderstandings. Things come off very differently than intended.” (P*#*4, 24 years old, Female, Master's Student)*

Communication challenges not only impede understanding but also amplify stress and reduce confidence in seeking professional support, highlighting the need for culturally and linguistically sensitive mental health services.

## Discussion

This exploratory qualitative study identified five main themes and 16 subthemes, covering mental health literacy, challenges faced, coping strategies, barriers to seeking professional help, and recommendations for improving support.

This study identified low to moderate mental health literacy among international university students, influenced by cultural norms, language barriers, and limited prior exposure to mental health education within the studied institutional context. These factors appear to be closely shaped by students’ sociocultural backgrounds and the absence of structured, culturally responsive mental health sensitisation programmes [24]. Similar patterns of low mental health literacy have been reported in other studies involving international student populations, suggesting that while the specific manifestations may be context-dependent, the underlying mechanisms, such as cultural framing of mental health and restricted access to tailored information, may extend to comparable settings [8,25]. Rather than implying universal generalizability, these findings highlight how institutional practices related to orientation, communication, and student engagement can influence mental health literacy among international students in similar higher education environments.

Stress and anxiety among international university students reflect a broader pattern of acculturative stress, where uncertainty, social disconnection, academic demands, and financial strain interact to heighten psychological distress [26]. Academic pressure is often intensified by unfamiliar pedagogical approaches and visa-related expectations, while financial instability further compounds stress exposure [27,28]. Limited host-country language proficiency can exacerbate social isolation, which has been consistently associated with elevated depressive symptoms [28]. Although substance use appears less central, it has been described in some contexts as a maladaptive coping response to sustained stress [29]. At the same time, evidence points to the moderating role of resilience factors, such as strong peer networks and institutional support, indicating that while stressors may vary across settings, the mechanisms influencing mental health may extend to other international education environments [27,29].

In the context of this institution, international university students primarily rely on peer support, self-coping strategies, and family connections, likely because these sources are more accessible, culturally familiar, and emotionally safe than formal services [30]. Peer-based practices, such as mindfulness, meditation, and social engagement, support shared understanding and personal agency [29,30], while family connections provide emotional continuity but are limited by physical distance [30,31]. Professional mental health services were less frequently utilised, reflecting context-specific barriers including low awareness, cultural stigma, language difficulties, and confidentiality concerns [24,25]. These findings suggest that while the reliance on informal support may be shaped by local institutional factors, the underlying mechanisms, social support, coping strategies, and perceived accessibility may be relevant in other international education contexts. NTU could strengthen mental health support by implementing culturally tailored, peer-led programmes, multilingual awareness campaigns, and structured orientation sessions to encourage help-seeking while respecting students’ social and cultural backgrounds.

Concerns about confidentiality and privacy can deter international students from seeking professional mental health support, likely because they perceive formal services as risky or unfamiliar, consistent with previous studies [25,31]. These fears often stem from potential record disclosure, discrimination, or academic and immigration consequences [31,32], and may be intensified by limited knowledge of local privacy regulations and cultural stigma [32]. When confidentiality is unclear, institutional support systems are often underutilized, whereas peer-led programmes and clear confidentiality policies can help build trust and encourage engagement [33]. Although strategies such as maintaining collectivist values in an individualistic context may aid coping with cultural differences, they do not fully address concerns about privacy or stigma [34]. NTU and similar universities can reduce these barriers by implementing culturally sensitive policies, safeguarding confidentiality, and fostering trust, thereby promoting help-seeking and supporting international students’ well-being and academic success.

Limited access to services and a lack of awareness are essential barriers for international students in seeking mental health services. The majority of students remain unaware of available resources or are faced with logistical, financial, and cultural impediments, aligning with other studies conducted in Australia and Singapore [25,31]. While some universities can't offer culturally responsive services, others are effective in promoting awareness through specialised counselling and peer support programmes [33,35]. Online platforms also offer a more accessible, culturally responsive option, although they may not fully replace the need for face-to-face support [36].

Stigma and cultural attitudes greatly discourage international students from accessing mental health services, consistent with other studies in Asia [24,31]. Mental health problems are misunderstood or stigmatised in most cultures, resulting in fear of judgment, discrimination, or weakness [24,26,35]. For instance, Asian students shy away from counselling because Confucian values stress emotional control and family honour [37]. Likewise, fear of visa status or academic performance further discourages students from accessing help [38]. There is also the cultural meaning of mental health involved. In some Middle Eastern and Asian cultures, distress is communicated in physical rather than mental health terms, and therefore, less recognition of the need to access professional services occurs [35]. These issues deter people from talking about issues openly and are accompanied by independence, elevating the likelihood that mental illness will intensify.

International university students often rely on individual coping strategies and self-reliance to manage mental health challenges, likely because these approaches are more accessible, culturally familiar, and perceived as safer than formal services, consistent with previous studies [30,31]. University students frequently avoid professional help due to stigma, privacy concerns, limited knowledge, or cultural norms, preferring to manage problems on their own [32,38]. Many employ personal resilience, peer or family support, and customary practices to reduce stress, which can be effective in the short term [37]. However, these strategies may not adequately address more severe or chronic conditions, such as depression or anxiety [30]. Overreliance on self-coping can delay professional intervention, potentially exacerbating mental health problems over time and highlighting the need for context-sensitive support mechanisms.

Misunderstandings or lack of awareness of mental health conditions pose significant barriers for international students, often delaying help-seeking, consistent with previous studies [31,37,38]. Many students interpret their symptoms as stress or pressures related to academics or finances, rather than indicators of a need for professional support [12,32]. Cultural stigma further complicates the issue, as mental health problems are often viewed as personal weaknesses rather than medical conditions [37]. For instance, an Australian study found that labelling mental illness as “craziness” discouraged students from seeking help [39]. In this context, culturally sensitive awareness sessions integrated into orientation and accessible campus-based guidance, could help students recognise mental health concerns and seek support in a safe and familiar setting.

Language barriers and cultural differences can hinder international students from seeking mental health support, contributing to misunderstandings, social isolation, and reduced use of available services, consistent with previous studies [24,31]. While multilingual counselling, cultural competence training for professionals, and technology-based tools have potential to mitigate these obstacles, evidence suggests that technology alone is insufficient, emphasizing the need for in-person, culturally sensitive support [9,39]. Locally, combining multilingual services, culturally responsive training for counsellors, and digital platforms for supplementary support could help bridge these gaps and improve access to and effectiveness of mental health care for international students.

### Limitations and mitigations

This study has several limitations. First, it involved 20 international university students from a single university in Nantong City, which may limit the transferability of the findings to other institutional or regional contexts. However, consistent with qualitative principles that prioritize information power over sample size, the dataset was sufficiently rich to support an in-depth exploration of participants’ experiences. Second, researcher subjectivity is an inherent consideration in qualitative research, as interpretations may be shaped by the researcher’s positioning. This was addressed through reflexive practices, including analytic memo writing, peer debriefing, and regular consultation with co-investigators to enhance analytic transparency. Third, participants’ accounts may have been influenced by social desirability bias, with some responses reflecting socially acceptable views. To reduce this risk, interviews were conducted in a confidential, non-judgmental manner using open-ended questions to encourage honest reflection. Future studies could strengthen transferability by involving more diverse international student populations across multiple universities and by incorporating mixed-methods designs to triangulate qualitative findings.

## Conclusion

International university students face multifaceted mental health challenges, including low mental health literacy, isolation, academic stress, stigma, and limited awareness of available services. Many depend on peer support and self-coping strategies while overlooking professional help, leading to delays in appropriate care. Addressing these barriers is essential to improving their well-being and academic outcomes. It is recommended that NTU and similar institutions strengthen confidentiality in mental health services through clear policies and proactive outreach. Mental health professionals working with NTU’s counselling services should receive continuous cultural sensitivity training, and accessibility should be expanded via orientation programmes and digital platforms. Offering multilingual counselling, peer support networks, and collaborations with local mental health providers will foster a more inclusive and supportive environment for international students.

### Key takeaways

i At Nantong University, international students face significant psychological challenges, including isolation, academic pressure, and financial stress.
ii Barriers to seeking professional help include stigma, lack of awareness, privacy concerns, and communication challenges.
iii Recommendations focus on building trust, improving cultural competence, enhancing accessibility, and providing institutional support to address these issues effectively.

## Supporting information

S1 File Selected qualitative quotes. (DOCX)

S2 File Interview Guide. (DOCX)
