# Correction: International university students’ perspectives on mental health and help-seeking behaviours in China: An exploratory qualitative study

**DOI:** 10.1371/journal.pmen.0000713

**Published:** 2026-08-28

**Authors:** Magnus Michael Sichalwe, Minjie Chu, Grace Tavengana, Manas Ranjan Behera, Abdul Basit

The fourth author's name is spelled incorrectly. The correct name is: Manas Ranjan Behera. The correct citation is: Sichalwe MM, Chu M, Tavengana G, Behera MR, Basit A (2026) International university students’ perspectives on mental health and help-seeking behaviours in China: An exploratory qualitative study. PLOS Ment Health 3(4): e0000559. https://doi.org/10.1371/journal.pmen.0000559
