# Supplementary material for: International university students’ perspectives on mental health and help-seeking behaviours in China: An exploratory qualitative study
Source: PLOS Ment Health. 2026 Apr 6;3(4):e0000559. doi: 10.1371/journal.pmen.0000559 (PMC13052886; doi:10.1371/journal.pmen.0000559)
Supplement: S1 File — (DOCX) [file pmen.0000559.s001.docx]

**INTERVIEW GUIDE**

**Title:** International Students’ Perspectives on Mental Health and Help-Seeking Behaviours at Nantong University, China: An Exploratory Qualitative Study

**PART 1**

**Demographic Information of the study participant**

| ID | Age | Gender | Country | Level Education of (Bachelor, Master’s, PhD) | Professional Major | Year of Study | Number of years being in China |
| --- | --- | --- | --- | --- | --- | --- | --- |
| P1 |  |  |  |  |  |  |  |
| P2 |  |  |  |  |  |  |  |
| P3 |  |  |  |  |  |  |  |
| P4 |  |  |  |  |  |  |  |
| P5 |  |  |  |  |  |  |  |
| P6 |  |  |  |  |  |  |  |
| P7 |  |  |  |  |  |  |  |
| P8 |  |  |  |  |  |  |  |
| P9 |  |  |  |  |  |  |  |
| P10 |  |  |  |  |  |  |  |
| P11 |  |  |  |  |  |  |  |
| P12 |  |  |  |  |  |  |  |
| P13 |  |  |  |  |  |  |  |
| P14 |  |  |  |  |  |  |  |
| P15 |  |  |  |  |  |  |  |
| P16 |  |  |  |  |  |  |  |
| P17 |  |  |  |  |  |  |  |
| P18 |  |  |  |  |  |  |  |
| P19 |  |  |  |  |  |  |  |
| P20 |  |  |  |  |  |  |  |

**PART II**

1. **Could you please explain to us what you know about mental health in your own words?**

**Probing Questions:**

1. ***Probe*:** What types of mental health challenges or difficulties do people commonly experience in their daily lives?
2. ***Probe*:** In your opinion, what factors might contribute to mental health problems?

***Can you tell us more*...**

1. *Probe*: What are the signs and symptoms that someone might be struggling with their mental health issues?

***Can you tell us more*...**

1. ***Probe*:** Why do you think mental health is often overlooked or misunderstood among students?
2. ***Probe*:** In what ways do you think mental health influences a person’s overall well-being?

**PART III**

1. **Exploring Mental Health Challenges**
2. ***Probe*:** What do you think are the most common mental health challenges faced by international students at Nantong University?

**Please tell us more…**

1. ***Probe*:** What are your views on how living in a new country may affect students’ mental health and well-being?
2. ***Probe*:** What role do academic pressures play in shaping mental health challenges for international students at Nantong University?
3. ***Probe*:** What are your perspectives on how language barriers may influence the emotional well-being of international students at Nantong University?
4. ***Probe*:** In what ways do financial challenges shape the mental health experiences of international students at Nantong University?
5. **PART IV**
6. **What will you do if you or your friend is struggling with mental health problems?**

**Probe:** Who will you talk to or ask for help?

What steps will you take to support yourself or your friend?

1. ***Probe*:** In your opinion, what prevents international students from seeking professional mental health support at Nantong University?
2. ***Probe*:** How do you think stigma or cultural beliefs might affect international students’ willingness to seek mental health support at Nantong University?
3. ***Probe*:** What role do you think peer and social support play in encouraging help-seeking?

-What role do you think peer and social support play in discouraging help-seeking?

1. ***Probe*:** What do you know about where to access mental health or counselling services when you or a colleague needs support?

-**Probe:** Do you think the availability of mental health services on campus is sufficient? Why or why not?

**5. PART V**

Please tell us what measures you think our university should implement to address the mental health needs of international students.

1. ***Probe*:** What would you suggest to improve the willingness of international students to seek help for mental health challenges?
2. ***Probe*:** How do you think cultural competency among mental health professionals at our university can improve help-seeking practices for international students?

**END**

**Do you have any questions, comments or additions or need clarification somewhere?**

5. SUMMARY (Interviewer summarizes the discussion)

Thank you for your valuable contribution to this research. Your insights will greatly enhance our understanding of mental health literacy and help-seeking behaviours among international students at Nantong University. Your input is crucial in improving support systems and fostering a more inclusive and supportive environment.
