# Supplementary material for: International university students’ perspectives on mental health and help-seeking behaviours in China: An exploratory qualitative study
Source: PLOS Ment Health. 2026 Apr 6;3(4):e0000559. doi: 10.1371/journal.pmen.0000559 (PMC13052886; doi:10.1371/journal.pmen.0000559)
Supplement: S2 File — (DOCX) [file pmen.0000559.s002.docx]

**International students’ perspectives on mental health and help-seeking behaviours in China: an exploratory qualitative study**

**Mental health literacy**

**Knowledge of the meaning of mental health**

“Okay, for me, mental health is the overall well-being of human beings. It's the way of thinking that deals with the coping of people's behaviours, their thinking, their emotions, and, yeah, their overall well-being. It's like the well-being of people's emotions, thoughts, etc., in the whole package. Yeah, it's mental health.”
*(P#11, 26 years old, Male, Master’s Student)*

“I’m not familiar with this topic, so I think it’s best to skip this question and move on to the next one.”
*(P#15, 27 years old, Male, Master’s Student)*

**Knowledge of common mental health problems**

“There are some anxiety disorders, like… what’s that one? There’s… is it OCD? Yes, OCD, something to do with people who like clean spaces. We call it OCD, Obsessive Compulsive Disorder. Yes, OCD, and PTSD. They’re also mood disorders, like depression, stress, and all. There’s this other condition… is it schizophrenia? Yeah! Schizophrenia.”
*(P#12, 22 years old, Male, Bachelor Student)*

“Mmh… there is the heart because I think when you stick too much information in your heart or you keep it for yourself you can have mental health issues, there is the brain, I think overthinking too is a form of mental health disturbance.”
*(P#1, 22 years old, Female, Master’s Student)*

**Knowledge of contributors to mental health problems**

“As I told you before… the main impact is your workplace or the environment that is around you. Like, if you are a student, it will be your university. If you are doing a job somewhere, it will be your office or wherever you are. If you are in the home, then it will be affected by the home as well. Moreover, it is also connected to the financial.”
*(P#7, 24 years old, Male, Master’s Student)*

“Mainly, I would say trauma. Be it family trauma, be it tragic trauma like accidents, be it multiple sorts of trauma, that's one. So the trauma, environment, isolation, being misunderstood.”
*(P#16, 24 years old, Female, Bachelor Student)*

**Perceived signs and symptoms of mental health problems**

“I think self-isolation is the biggest sign. I don't think I know any other ones. Apart from that bigger one, other small ones. People are talking less. Talking less. Talking less, going out less. All those things that eventually lead to self-isolation, I guess.”
*(P#4, 24 years old, Female, Master’s Student)*

“Some people just eat to try and cope, and also excessive substance use, whether alcohol or drugs… less confidence can also be a symptom, and unhealthy behaviour or bad actions too, these are signs, in my mind.”
*(P#19, 22 years old, Female, Bachelor Student)*

**Knowledge of the impact of mental health on well-being**

“Of course, if someone’s mental health is not okay, they might stop talking to others, skip meals, and lose their physical and mental stability. In such cases, what can they do in their life? They might even take their own life. They can commit suicide. As a human, if someone is not okay, they might end their life. This is the reality.”
*(P#8, 22 years old, Male, Bachelor Student)*

“Don't think so because it's not a big deal to overcome, so people can easily overcome their mental health sometimes. Because as I mentioned before, if I get homesickness, I just call my family members and talk with them. So I think it's easier to overcome this mental health, especially for me. It's my opinion.”
*(P#14, 28 years old, Male, Master’s Student)*

**Mental health-related challenges faced by international students**

**Social isolation and substance abuse**

“Judging from my experiences with going outside, I would say, especially the substance abuse, like excessive drinking. Also, for females, isolation is too much. You know, sometimes you just go outside and see a new face that you've never seen before, but you're told this person has been here for like two years or more, and you wonder why… yeah, the isolation, the substance abuse. And also, I think some financial issues. Yeah.”
*(P#6, 28 years old, Female, Master’s Student)*

**Stress and anxiety due to academic and financial pressures**

“I think anxiety… Many of us didn’t study in China before, so we’re not used to the environment. Even if you haven’t done anything wrong, constant notices about scholarship cancellations feel like daily threats. When I first arrived, I was extremely anxious, especially about opening WeChat, which was always full of negative news.”
*(P#5, 25 years old, Female, Master’s Student)*

**Approaches to managing mental health challenges**

**Reliance on peers, family, and social support**

“I will try to help by talking to her and helping her feel understood. Keeping things to ourselves can affect us a lot. If we create an opportunity to share, she might feel relief and less alone.”
*(P#1, 23 years old, Male, Master’s Student)*

“I talk to people from home. Yeah, I feel they're going to understand me better than I'll be understood here. Even in the matter of an emergency, I will still talk with people back home.”
*(P#18, 25 years old, Male, Master’s Student)*

**Limited reliance on professional help**

“I'm not a doctor to answer this question. But as a human, if it happens to me, I will go to the doctor and tell him the story. Maybe she or he can help me. And for my friend also, if I see this situation, I will invite him or I will take him to the doctor.”
*(P#6, 28 years old, Female, Master’s Student)*

**Barriers to seeking professional mental health support**

**Confidentiality and privacy concerns**

“We feel our privacy is always breached here. How do we know certain people have been called for therapy? It's already out there.”
*(P#6, 28 years old, Female, Master’s Student)*

“Our privacy is not protected that much. One way or the other, someone will know what you're going through.”
*(P#18, 25 years old, Male, Master’s Student)*

**Limited access to services and lack of awareness**

“I’m not sure I can pinpoint where, but I think it’s somewhere on campus here. Yeah, one of these offices, but I don’t know the exact location.”
*(P#13, 30 years old, Male, Master’s Student)*

“Firstly, we've been here for some months now and we've never even had any seminar on how to deal with these issues, even mental health issues.”
*(P#10, 25 years old, Male, Master’s Student)*

**Stigma and cultural perceptions**

“We need counselling because we’re here alone. But when we reach out, we’re told to ‘be careful’ and that they’re ‘not responsible.’”
*(P#8, 22 years old, Male, Bachelor Student)*

**Proactive and self-reliant coping strategies**

“If it is depression, firstly, I will try to find the reason for being depressed and understand what’s causing it. For mental health issues like depression, I can invite my friends to more social activities and try to focus on positive aspects of life, like exploring new things.”
*(P#3, 24 years old, Female, Bachelor Student)*

**Misunderstandings or lack of recognition of mental health issues**

“I think in social life, if students are stressed and they have depression, people see it as a normal thing. They think if you are a student, you have a hard life because you are struggling with studies or something. But I think it's completely wrong.”
*(P#3, 24 years old, Female, Bachelor Student)*

“Because when you're sick on the outside, when you're physically sick, you can see it. Someone can tell you your leg is broken. But when you're feeling heartache, no one can see that. It's just you. So they're not going to take things they can't see seriously.”
*(P#5, 25 years old, Male, Master’s Student)*

**Communication challenges**

“It’s confusing because they speak English, but it’s hard to understand their tone and meaning. The way they speak can sound harsh, even if it’s normal for them. Notices often seem translated from Chinese, leading to misunderstandings. Things come off very differently than intended.”
*(P#4, 24 years old, Female, Master’s Student)*
